# Supplementary material for: Using Regional Sero-Epidemiology SARS-CoV-2 Anti-S Antibodies in the Dominican Republic to Inform Targeted Public Health Response
Source: Trop Med Infect Dis. 2023 Nov 4;8(11):493. doi: 10.3390/tropicalmed8110493 (PMC10675152; doi:10.3390/tropicalmed8110493)
Supplement: Supplementary file 1 [file tropicalmed-08-00493-s001.zip › tropicalmed-2630548-supplementary.pdf]

Supplementary Materials:

Using Regional Sero-Epidemiology SARS-CoV-2 Anti-S Antibodies in the Dominican Republic to Inform Targeted Public Health Response

Beatris M. Martin [1,*], Angela Cadavid Restrepo [1], Helen Mayfield [1], Cecilia Then Paulino [2], Micheal de St. Aubin [3,4], William Duke [5], Petr Jarolim [3,6], Emily Zielinski Gutiérrez [7], Ronald Skewes Ramm [2], Devan Dumas [3,4], Salome Garnier [3,4], Marie Caroline Etienne [3], Farah Peña [2], Gabriela Abdalla [3], Beatriz Lopez [7], Lucia de la Cruz [2], Bernarda Henriquez [2], Margaret Baldwin [3,4], Benn Sartorius [1], Adam Kucharski [8], Eric J. Nilles [3,4,6] and Colleen L. Lau [1]

1 School of Public Health, Faculty of Medicine, The University of Queensland, Brisbane, QLD 4006, Australia; a.cadavidrestrepo@uq.edu.au (A.C.R.); h.mayfield@uq.edu.au (H.M.); b.sartorius@uq.edu.au (B.S.); colleen.lau@uq.edu.au (C.L.L.)

2 Ministry of Health and Social Assistance, Santo Domingo 10514, Dominican Republic; farahninoska@gmail.com (F.P.)

3 Brigham and Women's Hospital, Boston, MA 02115, USA; mgam81@gmail.com (G.A.); enilles@bwh.harvard.edu (E.J.N.)

4 Harvard Humanitarian Initiative, Cambridge, MA 02138, USA

5 Pedro Henriquez Urena National University, Santo Domingo, Dominican Republic; wduke@unphu.edu.do

6 Harvard Medical School, Boston, MA 02115, USA

7 Centers for Disease Control and Prevention, Central America Regional Office, Guatemala City 01015, Guatemala; fdx8@cdc.gov (B.L.)

8 London School of Hygiene & Tropical Medicine, London WC1E 7HT, UK; adam.kucharski@lshtm.ac.uk

* Correspondence: b.mariomartin@uq.net.au

Supplementary methods:

Survey weights

The weight of the selection probability was calculated in three stages. In the analysis reported in this study, the region (10 regions) and the area of residence (urban or rural setting) were combined to create 20 categories. The categories used here differ from the previous national report (1). In our previous report, the categories used to calculate the weights were defined based on the country's division into 5 areas, which were combined with the area of residence, resulting in 10 categories.

Here, firstly, the probability of a cluster being selected (p_c) was calculated based on the total number of clusters in each category (Supplementary Table 1), and the weight (w_c) was the inverse of the probability

of selection for each category ($w_c = 1/p_c$). Secondly, the probability of a household being selected (p_h) was calculated based on the total number of households in each cluster, and the weight (w_h) was the inverse of the probability of household selection ($w_h = 1/p_h$). Thirdly, the weights (w_f) from the first two steps were multiplied ($w_f = w_c * w_p$) and corrected for a finite population. The finite population was calculated as the sum of the probability of selection for each category and the probability of selection for each cluster.

Supplementary Table S1: Weights for Regions and setting categories based on probability of selection for each sampled cluster, Dominican Republic, June-October 2021.

Category	REGION	Setting	No. clusters per region	No. clusters selected	Selection probability	Weight
1	El Valle	Rural	1003	3	0.003	334.33
2	El Valle	Urban	124	4	0.032	31.00
3	Enriquillo	Rural	628	5	0.008	125.60
4	Enriquillo	Urban	297	10	0.034	29.70
5	Higuamo	Rural	1053	11	0.010	95.73
6	Higuamo	Urban	299	12	0.040	24.92
7	Metropolitana	Rural	234	9	0.038	26.00
8	Metropolitana	Urban	163	12	0.074	13.58
9	Nordeste	Rural	1203	5	0.004	240.60
10	Nordeste	Urban	329	5	0.015	65.80
11	Noroeste	Rural	836	4	0.005	209.00
12	Noroeste	Urban	208	3	0.014	69.33
13	Norte	Rural	1755	15	0.009	117.00
14	Norte	Urban	397	11	0.028	36.09
15	Sur	Rural	1131	4	0.004	282.75
16	Sur	Urban	286	5	0.017	57.20
17	Valdesia	Rural	1279	5	0.004	255.80
18	Valdesia	Urban	311	4	0.013	77.75
19	Yuma	Rural	823	4	0.005	205.75
20	Yuma	Urban	206	3	0.015	68.67

The age and gender distribution used/employed/adopted/incorporated to post-stratification (%):

Supplementary table S2: The Dominican Republic age and gender population distribution (2).

	5-14	15-24	25-34	35-44	45-54	55-64	65-74	≥75
**Male (0)**	9.980	9.731	7.745	6.256	4.617	3.128	1.787	1.440
**Female (1)**	9.831	9.384	7.646	6.008	5.263	3.476	1.837	1.440
**other (3)**	0.140	0.135	0.109	0.086	0.070	0.047	0.026	0.020

Supplementary results:

Anti-S prevalence, PT_{80} for the ancestral and Delta strains

Supplementary Table S3: National SARS-COV-2 anti-S antibody prevalence by demographic, COVID-19 vaccination status, and region, Dominican Republic, June-October 2021.

Covariates	Total participants, n	Seropositive participants, n	Adjusted[1] seroprevalence, % (95% CI)
Overall	6683	5958	85.4 (81.9-88.0)
Regions			
Yuma	330	265	78.7 (75.0-82.2)
Nordeste/Sur	533	476	86.6 (81.6-90.7)
Valdesia	489	429	83.3 (77.9-87.9)
Noroeste/El Valle	602	548	88.8 (83.6-92.8)
Norte	1306	1164	86.6 (73.5-94.8)
Higuamo	1717	1537	85.4 (79.7-90.1)
Metropolitana	999	897	84.1 (80.0-87.7)
Enriquillo	707	642	90.4 (86.1-93.8)
Gender			
Male	2494	2205	85.8 (81.6-90.0)
Female	4144	3712	85.0 (82.8-87.2)
Others	45	41	85.2 (71.2-99.2)
Age, years			
05-17	912	660	69.9 (65.9-73.7)
18-54	3795	3475	90.4 (85.9-93.9)
≥55	1976	1823	92.3 (88.3-95.4)
Area of residence			
Rural	3086	2682	82.4 (76.8-87.2)
Urban	3597	3276	87.2 (84.3-89.8)
No. household residents			
1-2	1427	1275	86.7 (77.9-93.1)
3-4	2776	2462	85.1 (80.7-88.9)
≥5	2480	2221	85.1 (82.6-87.3)
Work environment			
Outdoor	527	443	79.0 (65.1-89.4)
Indoor/ Mix	1531	1432	94.6 (92.1-96.5)
Smoking status			
Non-smoker	6214	5549	85.2 (82.2-88.1)

Current smoker	469	409	88.2 (81.7-94.7)
COVID-19 vaccine doses			
None	2576	1966	74.0 (68.4-79.1)
One dose	952	873	92.7 (90.6-94.5)
Two or more doses	3155	3119	98.9 (97.9-99.5)

[1]Adjusted for study design (selection probability, clustering), finite population correction, and post-stratified for age and sex.

Supplementary Table S4. Adjusted anti-S prevalence, correlates of protection and vaccine coverage by region in the Dominican Republic, June-October 2021.

	Anti-S adjusted prevalence	PT_{80} ancestral strain	PT_{80} Delta strain	Vaccine coverage[1]
Yuma	78.7 (75.0-82.2)	57.7 (54.6-60.9)	30.0 (27.8-32.2)	22.1 (16.7-28.1)
Nordeste/Sur	86.6 (81.6-90.7)	63.7 (58.4-68.7)	32.7 (27.3-38.4)	37.7 (30.4-45.3)
Valdesia	83.3 (77.9-87.9)	62.3 (61.2-63.3)	35.4 (30.6-40.4)	23.2 (19.1-27.6)
Noroeste/El Valle	88.8 (83.6-92.8)	65.6 (60.1-70.9)	31.6 (24.5-39.3)	35.9 (25.3-47.5)
Norte	86.6 (73.5-94.8)	65.2 (52.8-76.3)	40.6 (33.1-48.4)	37.3 (35.3-39.4)
Higuamo	85.4 (79.7-90.1)	70.1 (61.9-77.5)	38.3 (33.0-43.8)	41.5 (31.0-52.6)
Metropolitana	84.1 (80.0-87.7)	69.6 (63.2-75.5)	35.5 (31.3-39.9)	30.2 (23.8-37.1)
Enriquillo	90.4 (86.1-93.8)	72.0 (70.7-73.3)	43.0 (37.1-49.0)	40.1 (25.4-56.0)

[1]Two or more doses of vaccine coverage.

Supplementary Table S5. Adjusted anti-S prevalence, correlates of protection and vaccine coverage by cluster in the Dominican Republic, June-October 2021.

Cluster-ID	Provincia	Barrio/Paraje	Region	Anti-S (95% CI)	PT_{80} Ancestral (95% CI)	PT_{80} Delta (95% CI)	Vaccine Coverage[1] (95% CI)
A_01R	Santiago Rodriguez	La Leonor	Cibao Noroeste	82.5 (81.1-83.8)	66.8 (65.6-68.0)	40.8 (39.1-42.6)	20.1 (18.8-21.4)
A_02	Santiago Rodriguez	Las Caobas Adentro	Cibao Noroeste	85.8 (82.9-88.6)	59.4 (57.1-61.7)	29.6 (28.3-31.0)	29.6 (25.4-33.7)
A_03	Monte Cristi	La Judea	Cibao Noroeste	94.0 (93.2-94.9)	85.0 (83.0-86.9)	28.8 (26.2-31.3)	75.5 (73.5-77.6)
A_04	Monte Cristi	Guatapanal	Cibao Noroeste	89.1 (88.0-90.2)	38.6 (35.0-42.2)	6.8 (5.8-7.9)	14.1 (11.7-16.5)
A_05	Dajabon	La Ceiba	Cibao Noroeste	84.5 (81.3-87.6)	67.7 (66.1-69.3)	31.1 (30.1-32.1)	46.7 (40.3-53.1)
B_01	Duarte	El Vaticano	Cibao Nordeste	87.4 (86.6-88.1)	63.0 (62.0-64.0)	27.7 (27.0-28.3)	50.6 (48.7-52.5)
B_02	Duarte	La Madeja	Cibao Nordeste	92.0 (90.5-93.6)	72.2 (69.2-75.2)	25.8 (24.2-27.3)	28.4 (26.9-29.9)
B_03	Duarte	Urbanizacion Campo Fernandez I y II	Cibao Nordeste	82.3 (78.2-86.5)	35.2 (32.0-38.4)	17.5 (15.4-19.6)	47.7 (43.5-51.8)
B_04	Espaillat	Boca Ferrea Abajo (Aguacate)	Cibao Norte	88.2 (85.4-90.9)	65.9 (63.3-68.6)	33.5 (31.5-35.6)	59.0 (55.2-62.8)
B_05	Espaillat	La Encantada	Cibao Norte	83.0 (79.3-86.6)	56.1 (54.7-57.6)	26.2 (24.7-27.8)	54.0 (49.3-58.7)
B_06	Espaillat	Los Gonzalez	Cibao Norte	95.1 (94.4-95.9)	77.6 (76.5-78.7)	46.9 (44.5-49.3)	52.2 (46.8-57.7)
B_07R	Espaillat	Guayabillo	Cibao Norte	87.4 (86.0-88.9)	62.5 (60.6-64.4)	36.1 (35.1-37.2)	70.4 (67.9-72.9)
B_08	Espaillat	El Picacho	Cibao Norte	98.0 (97.2-98.7)	91.6 (90.9-92.3)	64.2 (62.5-65.8)	63.2 (60.3-66)
B_09	Espaillat	Los Pichardo	Cibao Norte	91.4 (90.5-92.2)	73.8 (72.7-74.9)	52.9 (52.0-53.8)	67.9 (66.1-69.6)
B_10	Espaillat	Canca La Reyna	Cibao Norte	86.8 (85.0-88.6)	66.0 (63.6-68.4)	38.2 (36.4-39.9)	52.2 (49.6-54.9)
B_11	Espaillat	El Higuerito Arriba	Cibao Norte	92.9 (91.8-93.9)	79.0 (78.2-79.9)	48.1 (46.7-49.6)	58.7 (57.5-59.9)
B_12R	Espaillat	Los Lopez	Cibao Norte	92.9 (90.9-95.0)	81.0 (79.6-82.5)	58.1 (55.9-60.2)	51.1 (46.3-55.8)
B_13	Espaillat	Manuel Rodríguez	Cibao Norte	93.6 (92.6-94.5)	72.1 (70.8-73.3)	46.6 (45.1-48.1)	53.2 (47.9-58.4)
B_14	La Vega	Santa Ana	Cibao Sur	96.2 (95.5-96.9)	75.1 (72.2-78.0)	44.1 (41.7-46.6)	63.5 (62.2-64.8)
B_15	La Vega	Rio Seco	Cibao Sur	86.9 (85.8-87.9)	64.3 (61.3-67.3)	33.4 (31.6-35.1)	41.9 (38.9-45.0)
B_16	La Vega	San Martin II	Cibao Sur	98.4 (97.9-98.9)	63.6 (61.5-65.6)	25.2 (22.4-28.0)	20.3 (19.0-21.7)
B_17	La Vega	Urbanización Doña Miledys	Cibao Sur	88.8 (87.3-90.4)	75.3 (70.7-79.9)	40.7 (37.3-44.0)	53.7 (50.8-56.7)
B_18	La Vega	El Centro Del Pueblo	Cibao Sur	78.0 (75.6-80.5)	50.9 (48.4-53.4)	29.7 (28.0-31.4)	46.2 (44.1-48.3)
B_19	Maria Trinidad Sanchez	Playa Navío	Cibao Nordeste	91.4 (90.3-92.4)	56.0 (53.4-58.6)	24.6 (23-26.2)	43.4 (41.8-45.0)

B_20	Puerto Plata	Gualetico	Cibao Norte	69.6 (67.4-71.8)	37.4 (34.5-40.3)	22.7 (20.7-24.7)	16.5 (14.5-18.5)
B_21	Hermanas Mirabal	El Abanico	Cibao Nordeste	*2	50.0 (46.0-54.1)	25.2 (23.1-27.3)	45.7 (42.4-48.9)
B_22	Sanchez Ramirez	La Bomba	Cibao Sur	*2	82.3 (79.8-84.9)	47.9 (45.6-50.3)	37.0 (34.5-39.6)
B_23	Sanchez Ramirez	Los Pinos	Cibao Sur	95.2 (94.3-96.2)	78.5 (77.0-80.0)	47.9 (46.5-49.4)	63.8 (62.6-64.9)
B_24	Santiago	Cienfuegos	Cibao Norte	92.0 (90.6-93.3)	71.6 (70.1-73.1)	36.1 (34.6-37.6)	20.9 (20.0-21.8)
B_25	Santiago	La Otra Banda	Cibao Norte	92.7 (91.6-93.9)	67.1 (62.7-71.5)	38.2 (34.6-41.8)	48.7 (43.0-54.3)
B_26	Santiago	Ensanche Bolivar	Cibao Norte	93.5 (92.8-94.2)	86.2 (84.9-87.5)	57.4 (55.2-59.7)	56.8 (54.7-58.9)
B_27R	Santiago	Los Jazmines	Cibao Norte	97.9 (97.7-98.2)	65.8 (61.6-70.1)	53.6 (51.2-56.0)	32.0 (30.4-33.5)
B_28	Santiago	Palmarito	Cibao Norte	81.6 (76.3-87.0)	66.0 (62.9-69.0)	34.5 (32.1-36.9)	43.2 (36.7-49.7)
B_29	Santiago	El Centro	Cibao Norte	72.6 (69.3-75.8)	56.2 (51.4-61.1)	23.5 (21.1-25.9)	28.2 (25.4-31.0)
B_30	Santiago	Villa Duarte Abajo	Cibao Norte	79.7 (78.8-80.5)	56.4 (54.0-58.7)	26.8 (24.9-28.7)	40.1 (38.2-42.0)
B_31	Santiago	Palma Picada de los Amaceyes	Cibao Norte	56.2 (52.9-59.4)	49.8 (46.1-53.5)	20.7 (17.3-24.0)	11.4 (10.0-12.8)
B_32R	Santiago	El Aguacate	Cibao Norte	54.8 (51.7-58.0)	37.8 (33.7-41.9)	16.0 (12.4-19.6)	5.2 (4.4-6.0)
B_33R	Santiago	Bernabe y Vereda De Amina	Cibao Norte	77.1 (74.9-79.3)	47.1 (43.0-51.2)	22.9 (16.8-28.9)	48.7 (43.9-53.5)
B_34	Santiago	El Piñón	Cibao Norte	71.7 (70.0-73.4)	50.8 (49.0-52.6)	18.9 (16.9-21.0)	9.3 (7.2-11.3)
B_35R	Santiago	Las Charcas y La Cruz	Cibao Norte	74.1 (71.5-76.7)	57.5 (55.3-59.7)	46.6 (43.7-49.5)	36.8 (33.3-40.2)
B_36	Santiago	San Jose Adentro	Cibao Norte	83.9 (81.1-86.7)	36.6 (34.9-38.3)	21.9 (20.2-23.7)	8.9 (8-9.9.0)
B_37	Santiago	Cuesta Arena y Sabana Grande De Batey I	Cibao Norte	62.6 (58.0-67.1)	32.9 (30.0-35.9)	17.5 (15.9-19.1)	30.3 (27.3-33.3)
B_38	Santiago	Jacagua	Cibao Norte	78.1 (74.8-81.3)	69.5 (66.5-72.6)	34.2 (30.1-38.4)	32.6 (28.9-36.2)
B_39	Valverde	La Altagracia	Cibao Noroeste	*2	72.1 (70.0-74.1)	29.0 (27.3-30.8)	26.7 (24-29.3)
B_40	Valverde	Sito Toro	Cibao Noroeste	93.7 (92.9-94.6)	54.0 (51.8-56.2)	28.9 (27.6-30.1)	37.7 (35.1-40.3)
B_41R	Monseñor Nouel	Los Quemados	Cibao Sur	84.1 (81.7-86.5)	69.5 (66.0-73.1)	56.7 (53.4-59.9)	42.7 (38.8-46.5)
B_42	Monseñor Nouel	San Pablo	Cibao Sur	85.8 (84.5-87.0)	56.6 (53.1-60.2)	26.1 (24.2-27.9)	32.9 (30.1-35.7)
C_01	Elias Piña	El Batey	El Valle	89.4 (86.3-92.6)	81.1 (78.7-83.5)	66.7 (65.5-67.8)	38.5 (34.5-42.5)
C_02	Barahona	Palo Bonito	Enriquillo	75.7 (74.4-77.0)	69.9 (69.2-70.5)	40.6 (38.2-43.1)	8.2 (7.7-8.8)
C_03	Barahona	El Higuito	Enriquillo	98.9 (98.5-99.3)	68.2 (66.2-70.2)	49.0 (46.2-51.8)	50.2 (41.0-59.4)
C_04	Barahona	El Majagual	Enriquillo	92.9 (91.9-93.9)	79.0 (77.6-80.4)	43.9 (43.0-44.8)	53.7 (47.2-60.2)
C_05R	Baoruco	Las Cañitas	Enriquillo	83.4 (80.6-86.2)	51.7 (49.9-53.5)	35.0 (33.8-36.2)	13.7 (12.7-14.7)

C_06R	Azua	Los Frios Almedio	Valdesia	94.7 (94.0-95.5)	66.8 (64.1-69.6)	15.8 (14.3-17.2)	35.8 (33.2-38.4)
C_07	San Juan	Guatapanal	El Valle	87.7 (85.1-90.4)	72.1 (70.0-74.1)	27.5 (26.2-28.9)	43.9 (38.3-49.4)
C_08	San Juan	Montes del Estado	El Valle	71.1 (65.9-76.3)	57.6 (54.1-61.1)	23.9 (22.9-25.0)	27.3 (23.7-30.8)
C_09	Independencia	Batey 9	Enriquillo	94.8 (94.0-95.6)	80.2 (78.9-81.4)	46.0 (45.0-46.9)	22.2 (18.8-25.5)
C_10R	Independencia	Tierra Nueva	Enriquillo	92.3 (90.0-94.5)	60.2 (57.4-63.0)	23.8 (22.2-25.3)	35.3 (33.9-36.7)
C_11	Azua	Santa Ana	Valdesia	94.9 (92.7-97.2)	79.2 (77.1-81.3)	40.2 (36.9-43.4)	62.1 (59.9-64.2)
C_12	Barahona	Blanquizales	Enriquillo	97.7 (97.2-98.2)	80.3 (77.5-83.0)	54.5 (51.3-57.7)	62.1 (57.9-66.3)
C_13	Barahona	Los Blocks	Enriquillo	82.4 (78.2-86.7)	71.8 (69.0-74.7)	45.4 (43.5-47.3)	62.2 (58.8-65.6)
C_14	Baoruco	Hato Nuevo	Enriquillo	98.1 (97.5-98.8)	90.3 (89.0-91.6)	63.3 (60.4-66.1)	84.3 (83.0-85.7)
C_15	Barahona	La Yagua o La Caya	Enriquillo	91.1 (90.0-92.1)	73.8 (71.6-75.9)	49.8 (47.0-52.5)	23.5 (21.2-25.8)
C_16	Pedernales	Los Cayucos	Enriquillo	84.2 (79.4-88.9)	73.4 (70.7-76.1)	39.2 (35.7-42.6)	54.3 (46.8-61.7)
C_17	Independencia	La Q	Enriquillo	84.9 (83.4-86.3)	69.9 (67.9-72.0)	35.4 (31.7-39.2)	31.7 (28.2-35.3)
C_18	Independencia	Brisas del Lago	Enriquillo	89.4 (87.0-91.8)	82.9 (80.5-85.4)	56.5 (52.4-60.6)	37.2 (33.1-41.3)
C_19	Baoruco	Las Malvinas	Enriquillo	84.8 (82.8-86.9)	56.1 (54.2-58.1)	41.1 (39.1-43.0)	28.7 (26.4-31.0)
C_20	Baoruco	Cabeza de Toro	Enriquillo	81.0 (75.1-86.9)	62.9 (59.2-66.5)	37.9 (36.9-38.8)	51.2 (44.8-57.5)
C_21	San Juan	La Mina	El Valle	85.8 (83.3-88.3)	73.3 (70.6-75.9)	47.3 (45.9-48.6)	26.1 (21.6-30.5)
C_22	San Juan	Centro del Pueblo	El Valle	87.4 (84.3-90.4)	64.5 (61.3-67.6)	33.9 (28.8-39.1)	33.6 (26.6-40.6)
C_23	Elias Piña	Centro de la Ciudad	El Valle	83.4 (78.0-88.8)	66.7 (62.7-70.6)	40.3 (36.3-44.3)	52.8 (46.9-58.7)
C_24	Elias Piña	Cristo Salvador	El Valle	82.9 (77.5-88.3)	64.9 (61.6-68.3)	47.9 (46.5-49.3)	43.5 (38.9-48.1)
D_01	Azua	Los Tramojos	Valdesia	88.9 (87.7-90.2)	67.4 (65.5-69.3)	41.8 (39.3-44.4)	19.6 (15.2-24.1)
D_02	San Cristobal	Sainagua	Valdesia	81.9 (79.1-84.7)	58.3 (55.4-61.2)	37.4 (32.2-42.6)	19.4 (15.9-22.9)
D_03R	San Jose de Ocoa	La Cruz y Los Palmaritos	Valdesia	*2	69.3 (65.7-72.8)	59.1 (55.8-62.5)	71.1 (64.6-77.6)
D_04R	Distrito Nacional	30 De Mayo	Metropolitana	97.7 (97.0-98.4)	93.6 (92.4-94.7)	62.9 (58.8-66.9)	36.5 (33.8-39.2)
D_05R	Distrito Nacional	Mata Hambre	Metropolitana	89.9 (87.3-92.6)	50.6 (48.8-52.3)	21.7 (19.8-23.7)	26.1 (21.4-30.7)
D_06R	Distrito Nacional	Julieta Morales (Los Praditos)	Metropolitana	98.3 (98.0-98.6)	85.7 (84.8-86.6)	52.6 (51.2-54.0)	28.1 (26.4-29.7)
D_07	Distrito Nacional	Simon Bolivar	Metropolitana	93.8 (92.6-95.0)	79.1 (77.4-80.9)	40.4 (36.4-44.4)	40.0 (32.5-47.4)
D_08R	Distrito Nacional	La Isabela	Metropolitana	90.2 (88.5-92.0)	68.9 (67.2-70.6)	24.2 (21.8-26.6)	26.8 (21.8-31.9)
D_09	Distrito Nacional	Ensanche la Fe	Metropolitana	*2	95.1 (94.0-96.3)	58.9 (56.1-61.8)	37.8 (31.9-43.7)
D_10	Santo Domingo	Los Paredones	Metropolitana	80.2 (75.2-85.3)	54.5 (49.2-59.8)	37.5 (34.8-40.2)	21.7 (19.5-23.9)

D_11R	Monte Plata (Borde Santo Domingo)	El Talado (El Talao)	Higuamo	79.1 (73.5-84.8)	54.6 (50.4-58.8)	29.8 (26.5-33.0)	46.3 (41.8-50.7)
D_12R	Santo Domingo	La Culata y San Joaquin	Metropolitana	84.7 (80.5-88.9)	58.9 (52.8-64.9)	22.1 (18.6-25.6)	42.0 (34.6-49.3)
D_13	Santo Domingo	Los Mameyes	Metropolitana	67.5 (60.6-74.3)	41.1 (39.7-42.5)	10.5 (9.3-11.8)	14.4 (10.1-18.8)
D_14	Santo Domingo	Batey Guanuma	Metropolitana	79.0 (74.9-83.0)	60.0 (57.8-62.3)	25.3 (20.7-29.8)	15.2 (12.0-18.3)
D_15	Santo Domingo	La Ceiba	Metropolitana	85.5 (84.3-86.8)	61.3 (57.9-64.6)	36.1 (34.8-37.4)	37.8 (32.9-42.8)
D_16R	Santo Domingo	El Dajao y Palmilla	Metropolitana	78.0 (73.2-82.9)	59.2 (57.4-61.0)	24.3 (20.3-28.4)	30.9 (25.3-36.4)
D_17	Santo Domingo	La Jacagua	Metropolitana	85.0 (80.8-89.1)	74.7 (71.5-77.9)	22.7 (21.2-24.3)	31.5 (25.9-37.1)
D_18	Santo Domingo	Mata Gorda	Metropolitana	80.9 (78.8-82.9)	67.0 (65.4-68.6)	34.1 (33.5-34.7)	25.3 (21.4-29.2)
D_19	Santo Domingo	Buenas Noches	Metropolitana	89.1 (88.3-89.8)	60.1 (57.1-63.1)	37.0 (33.3-40.6)	29.6 (24.7-34.5)
D_20	Sanchez Ramirez	El Cacao	Cibao Sur	96.9 (96.2-97.7)	73.7 (72.5-74.9)	31.6 (30.0-33.2)	47.0 (44.4-49.6)
D_21	San Pedro de Macoris	Batey Aleman	Higuamo	91.3 (88.6-94.1)	76.1 (73.4-78.7)	46.0 (44.0-47.9)	37.3 (34.1-40.5)
D_22	San Pedro de Macoris	Batey Campiña	Higuamo	85.6 (81.5-89.7)	70.9 (66.0-75.7)	40.4 (37.2-43.6)	26.7 (24.6-28.8)
D_23R	San Pedro de Macoris	Batey Monte Cristi	Higuamo	91.8 (88.9-94.7)	72.9 (71.3-74.4)	49.5 (48.0-51.0)	39.6 (37.3-41.8)
D_24	San Pedro de Macoris	Honduras	Higuamo	83.3 (79.8-86.8)	58.9 (57.1-60.8)	19.1 (16.8-21.5)	38.3 (32.6-44.0)
D_25	Samana	Agua Sabrosa	Cibao Nordeste	52.8 (49.9-55.8)	37.5 (35.2-39.9)	19.0 (16.9-21.1)	19.0 (17.6-20.3)
D_26R	Samana	La Colmena	Cibao Nordeste	67.8 (64.9-70.7)	28.3 (25.5-31.1)	1.0 (0.7-1.3)	*[3]
D_27	Azua	El Golfo	Valdesia	90.0 (89.1-91.0)	42.1 (40.0-44.2)	21.9 (20.5-23.3)	32.1 (30.3-33.8)
D_28	Monte Plata	Cuarce (Cuance)	Higuamo	34.3 (32.6-36.0)	10.5 (9.2-11.7)	5.4 (4.4-6.4)	*[3]
D_29	Monte Plata	La Piña	Higuamo	63.0 (59.7-66.3)	48.7 (47.2-50.3)	13.6 (12.3-14.8)	12.3 (10.3-14.2)
D_30R	Monte Plata	Coca Barrera	Higuamo	66.5 (59.5-73.6)	37.4 (31.8-42.9)	23.3 (19.8-26.8)	25.3 (19.9-30.7)
D_31	Hato Mayor	Rincon Bellaco	Higuamo	61.3 (56.5-66.1)	49.4 (46.5-52.3)	24.3 (22.1-26.5)	30.5 (26.7-34.3)
D_32	Maria Trinidad Sanchez	La Colmenas	Cibao Nordeste	84.8 (83.2-86.3)	68.8 (67.6-70.1)	26.6 (23.5-29.7)	14.5 (13.5-15.4)
D_33	Azua	Enriquillo	Valdesia	71.4 (65.9-76.9)	55.7 (51.6-59.8)	39.0 (38.1-40.0)	26.7 (20.9-32.5)
D_34	La Romana	Cucama	Yuma	81.3 (76.2-86.3)	52.4 (47.8-57.0)	24.8 (22.3-27.3)	23.0 (19.1-26.9)
D_35	San Pedro de Macoris	Guachupita	Higuamo	91.7 (91.0-92.4)	80.6 (79.6-81.6)	51.6 (49.5-53.6)	58.4 (56.6-60.2)

D_36	San Pedro de Macoris	Mexico	Higuamo	84.2 (82.0-86.5)	75.2 (73.4-77.1)	39.7 (36.9-42.5)	49.9 (43.8-56.0)
D_37	San Pedro de Macoris	Las Piedras	Higuamo	91.0 (89.3-92.7)	73.8 (72.2-75.5)	38.0 (36.6-39.3)	45.3 (38.3-52.3)
D_38	San Pedro de Macoris	Villa Magdalena	Higuamo	91.3 (89.5-93.0)	74.2 (73.0-75.5)	41.0 (39.9-42.1)	45.4 (40.3-50.5)
D_39	San Pedro de Macoris	El Brisal	Higuamo	88.2 (86.0-90.4)	73.9 (72.9-74.9)	43.9 (42.7-45.2)	31.5 (28.7-34.3)
D_40	San Pedro de Macoris	San Anton	Higuamo	84.5 (82.2-86.7)	65.9 (64.6-67.2)	40.9 (39.0-42.7)	38.3 (35.7-40.9)
D_41	San Pedro de Macoris	La Punta Pescadora	Higuamo	86.8 (84.4-89.3)	65.5 (64.2-66.9)	42.2 (40.4-44.0)	34.8 (31.8-37.8)
D_42	San Pedro de Macoris	Colina II	Higuamo	93.7 (92.3-95.2)	77.6 (75.0-80.2)	44.9 (42.7-47.0)	49.1 (46.0-52.2)
D_43	San Pedro de Macoris	El Patio	Higuamo	95.4 (94.9-95.9)	79.5 (77.4-81.6)	35.6 (34.0-37.3)	51.6 (49.0-54.3)
D_44	Monte Plata	La Madama	Higuamo	92.8 (91.9-93.7)	82.2 (80.9-83.6)	27.6 (24.8-30.4)	34.0 (32.8-35.2)
D_45	Monte Plata	30 De Mayo	Higuamo	86.2 (84.5-87.8)	68.9 (67.4-70.3)	39.9 (36.2-43.5)	55.3 (50.5-60.2)
D_46	Monte Plata	El Suizo	Higuamo	92.2 (90.7-93.6)	78.3 (77.1-79.5)	55.5 (54.0-56.9)	60.8 (57.2-64.3)
D_47	Maria Trinidad Sanchez	Ensanche José Ramírez	Cibao Nordeste	97.4 (96.9-98.0)	83.6 (82.0-85.3)	46.2 (42.8-49.7)	19.0 (16.9-21.1)
D_48	Santo Domingo	Santa Rosa	Metropolitana	69.8 (65.7-73.8)	59.5 (58.3-60.8)	30.9 (30.1-31.7)	17.7 (11.8-23.6)
D_49	San Cristobal	Guachupita/ Los Cocos (Kilómetro 28)	Valdesia	95.0 (93.9-96.2)	78.7 (75.9-81.4)	39.2 (33.2-45.1)	48.2 (41.9-54.5)
D_50	San Cristobal	Nigua Sur I	Valdesia	80.7 (79.8-81.6)	65.6 (63.5-67.7)	33.1 (31.3-34.9)	14.6 (13.4-15.7)
D_51	Santo Domingo	La Rosa	Metropolitana	94.3 (93.0-95.7)	78.5 (74.3-82.7)	28.8 (24.6-33.0)	43.0 (39.3-46.7)
D_52R	Distrito Nacional	Los Restauradores	Metropolitana	98.5 (98.2-98.8)	95.5 (94.7-96.2)	64.5 (61.5-67.6)	49.7 (43.9-55.5)
D_53R	Santo Domingo	Los Trinitarios y Mendoza	Metropolitana	73.2 (66.8-79.7)	51.6 (45.2-57.9)	27.8 (26.4-29.3)	27.3 (20.9-33.6)
D_54	Santo Domingo	San Jose de Mendoza	Metropolitana	71.5 (65.0-77.9)	60.2 (56.3-64.2)	18.4 (14.6-22.1)	26.2 (21.2-31.3)
D_55	Peravia	El Mani	Valdesia	64.8 (58.0-71.5)	39.8 (34.3-45.3)	14.4 (11.9-17.0)	13.5 (10.2-16.7)
D_56	Samana	El Almendro	Cibao Nordeste	87.0 (85.9-88.2)	64.4 (63.2-65.6)	19.1 (16.8-21.5)	14.8 (12.8-16.9)
E_01	Hato Mayor	Yanigua	Higuamo	53.2 (51.2-55.2)	37.7 (35.9-39.6)	13.3 (12.0-14.5)	9.3 (7.4-11.2)
E_02	El Seibo	Cabirmal	Yuma	54.4 (50.3-58.5)	30.8 (28.5-33.1)	17.5 (16.1-18.9)	5.4 (4.4-6.3)
E_03	El Seibo	Arroyo Lucas Campo	Yuma	58.5 (56.3-60.7)	36.6 (35.1-38.1)	17.0 (16.0-18.0)	6.5 (5.3-7.6)
E_04	La Altagracia	San Pedro	Yuma	77.4 (76.2-78.7)	60.9 (59.1-62.7)	34.3 (32.2-36.4)	24.6 (21.4-27.9)

E_05	El Seibo	El Matadero	Yuma	88.4 (87.5-89.3)	57.8 (54.3-61.4)	14.2 (12.3-16.2)	36.7 (32.0-41.4)
E_06	La Altagracia	Batey Palo Bonito	Yuma	95.6 (95.1-96.0)	91.1 (90.2-92.0)	50.2 (47.7-52.7)	25.4 (23.6-27.2)
E_07	La Altagracia	Batey Maragua	Yuma	68.3 (63.2-73.3)	44.4 (40.9-47.9)	23.4 (22.0-24.7)	12.9 (10.6-15.2)

[1]Two or more doses of vaccine coverage. *[2]Estimated prevalence and 95% CI were not calculated once all participants from these clusters were anti-S positive. *[3]Estimated vaccine coverage and 95% CI were not calculated once no participants from these clusters had received two or more doses of vaccine against COVID-19.

Variable selection and Logistic Regression

Correlation between variables was detected for age group and work environment, age group and smoking status, gender and age group, gender and area of residence, gender and number of household members, gender and work environment, gender and smoking status, gender and number of vaccine doses, area of residence and work environment, area of residence and number of vaccine doses, number of household members and work environment, smoking status and work environment, and work environment and number of vaccines doses. When the multivariable model and the model with interaction terms for each pair of variables listed above were compared, the results of the likelihood test were significant for age and work environment, age and smoking status, gender and area of residence, gender and work environment, number of household members and work environment, and smoking status and work environment. Testing the models using the VIF test found no significant multicollinearity in the multivariable model; thus, the results reported here are from the multivariable model.

Multivariable National model

A higher OR for anti-S positivity was associated with five or more people per household (OR 1.98; 95% CI 1.06-3.39) compared to 1-2 people per household; indoor and mix of indoor/outdoor compared to outdoor work environment (OR 5.04; 95% CI 2.25-11.29); tertiary/technical compared to none/primary/secondary educational level (OR 1.52; 95% CU 1.15-2.01), and receiving one (OR 3.14; 95% CI 1.94-5.10) and two or more (OR 18.87; 95% CI 11.33-31.42) doses of vaccine against COVID-19 compared to unvaccinated participants. A lower OR for anti-S positivity was associated with participants being younger than 18 years old, compared to those 18-54 years old.

A higher OR for PT80 for the ancestral strain was associated with five or more people per household (OR 1.92; 95% CI 1.28-2.90) compared to 1-2 people per household, indoor and mix

of indoor/outdoor compared to outdoor work environment (OR 2.45; 95% CI 1.85-3.24), and receiving one (OR 1.71; 95% CI 1.12-2.61) and two or more (OR 2.62; 95% CI 1.93-3.55) doses of vaccine against COVID-19 compared to unvaccinated participants.

A higher OR for PT80 for the Delta strain was associated with indoor and mix of indoor/outdoor compared to outdoor work environment (OR 1.78; 95% CI 1.25-2.83) and receiving one (OR 2.23; 95% CI 1.68-2.95) or two or more (OR 2.11; 95% CI 1.52-2.92) doses of vaccine against COVID-19 compared to unvaccinated participants.

Multilevel National model

Supplementary Table S6. National level OR for PT80 for the ancestral and Delta strains from multilevel logistic regression, the Dominican Republic, June-October 2021.

	**Anti-S**		**Ancestral PT_{80}**		**Delta PT_{80}**	
	**OR**	**(95% CI)**	**OR**	**(95% CI)**	**OR**	**(95% CI)**
**Gender**						
Female (ref.)						
Male	0.63	(0.38-1.06)	1.51	(0.84-2.70)	1.07	(0.76-1.50)
Other	1.34	(0.83-2.17)	0.7	(0.16-3.09)	1.42	(0.62-3.27)
**Age, years**						
05-17	**0.17**	**(0.07-0.40)**	0.65	(0.36-1.18)	1.22	(0.69-2.18)
18-54 (ref.)						
≥55	1.52	(0.85-2.72)	1.27	(0.77-2.09)	**1.45**	**(1.11-1.89)**
**Educational Level**						
None/Primary/ Secondary (ref.)						
Tertiary/Technical	**1.78**	**(1.39-2.29)**	0.77	(0.50-1.19)	0.97	(0.71-1.32)
**Socioeconomic score, 0-5**						
0						
1-5 (ref.)	1.36	(0.32-1.7)	0.97	(0.34-2.77)	1.39	(0.56-3.42)
**Setting**						
Urban (ref.)						
Rural	1.33	(0.89-1.98)	1.23	(0.91-1.67)	1.31	(0.97-1.77)
**Household members, number of people/household**						
1-2 (ref.)						
3-4	1.28	(0.66-2.49)	1.18	(0.75-1.85)	1.04	(0.61-1.78)
≥5	2.74	(0.66-11.39)	3.41	(0.78-14.83)	1.11	(0.71-1.74)
**Work environment**						
Outdoor (ref.)						
Indoor/Mix	**9.49**	**(1.33-67.85)**	**4.34**	**(2.43-7.76)**	**2.41**	**(1.16-4.97)**
**Smoking status**						

Non-smoker (ref.)						
Current smoker	0.82	(0.42-1.58)	0.52	(0.26-1.02)	0.77	(0.42-1.40)
**Risk Factors**						
None	1.58	(0.85-2.92)	1.19	(0.69-2.06)	0.81	(0.05-1.33)
One or more (ref.)						
**Vaccine doses**						
Unvaccinated (ref.)						
One dose	**4.80**	**(2.35-9.83)**	**2.61**	**(1.72-3.94)**	3.89	(0.93-4.4)
Two or more doses	**85.94**	**(10.95-674.33)**	**4.78**	**(2.15-10.62)**	**3.08**	**(1.57-9.65)**

Multilevel regional models

Supplementary Table S7. Regional OR for Anti-S from regional multilevel logistic regression, the Dominican Republic, June-October 2021.

	Yuma		Higuamo		Nordeste/Sur		Noroeste/ El Valle		Norte		Valdesia		Enriquillo		Metropolitana	
	OR	95% CI	OR	95% CI	OR	95% CI	OR	95% CI	OR	95% CI	OR	95% CI	OR	95% CI	OR	95% CI
**Gender**																
Female (ref.)																
Male	0.57	0.44-0.87	0.54	0.14-2.06	2.75	0.22-34.34	0.44	0.15-1.32	0.57	0.3-1.06	1.04	0.26-4.08	0.88	0.41-1.86	0.64	0.31-1.31
Other	*		*		*		*		*		*		*		*	
**Age, years**																
05-17	0.27	0.08-0.55	0.11	0.05-0.25	0.25	0.04-1.57	0.04	0.002-0.8	0.27	0.08-0.85	0.51	0.26-1.04	0.31	0.09-1.08	0.37	0.18-0.76
18-54 (ref.)																
≥55	1.16	0.18-1.23	1.35	0.59-3.1	2.24	0.17-29.22	3.56	0.83-15.32	1.16	0.31-4.37	2.65	0.83-8.5	1.13	0.29-4.48	0.97	0.42-2.24
**Educational Level**																
None/Primary/ Secondary (ref.)																
Tertiary/Technical	2.21	0.83-2.4	1.12	0.57-2.23	14.83	1.95-112.71	10.02	0.24-410.08	2.21	0.51-9.55	3.28	0.96-11.17	1.34	0.39-4.54	1.35	0.51-3.57
**Socioeconomic score**																
0	12.80	0.17-1.27	0.68	0.27-1.74	1.75	0.18-16.89	0.44	0.04-4.98	12.80	0.61-268.93	3.00	0.52-17.21	0.52	0.19-1.44	0.52	0.24-1.13
1-5 (ref.)																
**Setting**																
Urban (ref.)																
Rural	1.50	0.21-2.06	1.37	0.46-4.03	1.42	0.3-6.75	5.24	0.65-42.06	1.50	0.61-3.71	0.82	0.23-2.99	0.38	0.2-0.73	1.80	0.72-4.52
**Household members**																
1-2 (ref.)																
3-4	1.61	0.18-2.99	2.14	0.72-6.4	0.19	0.02-1.74	0.47	0.12-1.89	1.61	0.66-3.93	0.33	0.16-0.68	1.04	0.38-2.86	1.38	0.71-2.67
≥5	6.66	0.87-3.48	2.85	0.77-10.51	0.75	0.12-4.76	0.21	0.03-1.32	6.66	2.29-19.42	0.19	0.12-0.31	0.64	0.17-2.41	3.28	1.43-7.54
**Work environment**																
Outdoor (ref.)																
Indoor/Mix	45.85	0.78-17.92	6.80	1.06-43.65	31.33	1.35-724.81	0.27	0.02-4.3	45.85	4.3-489.14	29.66	2.94-299.16	1.00	0.09-11.45	0.54	0.16-1.81
**Smoking status**																
Non-smoker (ref.)																
Current smoker	1.01	0.21-1.73	3.88	0.63-23.81	1.38	0.04-45.95	0.08	0.01-0.44	1.01	0.19-5.47	0.50	0.26-0.94	2.09	0.8-5.49	1.63	0.28-9.52
**Risk Factors**																
None	1.52	6.18-259.17	2.25	1.08-4.69	4.35	0.59-31.97	1.87	0.98-3.56	1.52	0.29-7.99	1.06	0.4-2.83	1.61	0.89-2.92	0.93	0.56-1.54
One or more (ref.)																
**Vaccine doses**																
Unvaccinated (ref.)																
One dose	3.79	1.22-11.59	2.99	1.13-7.93	10.54	1.19-93.11	3.33	0.66-16.78	3.79	1.02-14	1.87	0.43-8.16	12.60	0.72-219.51	4.38	1.28-15.01
Two or more doses	46.04	1.29-8.7	25.05	11.76-53.35	1494.60	57.64-38753.64	55.88	2.06-1519.24	46.04	9.85-215.19	45.77	9.24-226.7	18.86	7.59-46.9	23.08	4.57-116.66

*Due to the small sample size, results were not calculated.

Supplementary Table S8. Regional OR for PT80 for ancestral strain from multilevel logistic regression, the Dominican Republic, Jun-Oct 2021.

	Yuma		Higuamo		Nordeste/Sur		Noroeste/ El Valle		Norte		Valdesia		Enriquillo		Metropolitana	
	OR	95% CI	OR	95% CI	OR	95% CI	OR	95% CI	OR	95% CI	OR	95% CI	OR	95% CI	OR	95% CI
**Gender**																
Female (ref.)																
Male	1.37	1.01-1.85	0.63	0.25-1.58	2.64	0.85-8.2	1.32	0.42-4.17	1.44	0.54-3.86	1.9	1.5-2.4	0.89	0.59-1.35	0.75	0.48-1.16
Other	1.34	0.05-38.63	1.51	0.22-10.25	*	*	*	*	60.33	0.12-29678.55	0.42	0.08-2.27	*	*	71.27	5.95-853.94
**Age, years**																
05-17	0.5	0.19-1.35	0.52	0.25-1.06	0.13	0.01-1.36	0.36	0.12-1.09	1.14	0.14-9.44	1.41	0.67-2.99	1.76	0.83-3.73	0.51	0.24-1.08
18-54 (ref.)																
≥55	0.49	0.24-1.01	0.76	0.51-1.11	1.66	0.56-4.88	1.77	0.41-7.71	13.14	0.46-372.36	1.18	0.78-1.79	1.99	0.71-5.55	1.18	0.7-1.98
**Risk Factors Level**																
None/Primary/ Secondary (ref.)																
Tertiary/Technical	0.67	0.33-1.38	0.87	0.53-1.43	0.72	0.34-1.56	5.95	0.83-42.87	0.05	0.01-0.31	1.08	0.62-1.88	1.15	0.64-2.1	0.7	0.32-1.54
**Socioeconomic score**																
0	1.63	0.47-5.66	1.05	0.49-2.25	2.05	0.79-5.35	1.68	0.15-18.69	0.32	0.01-19.84	4.08	1.25-13.3	1.25	0.41-3.8	0.25	0.09-0.7
1-5 (ref.)																
**Setting**																
Urban (ref.)																
Rural	0.59	0.18-1.87	1.76	0.87-3.56	1.39	1.08-1.8	0.32	0.11-0.94	0.2	0.09-0.43	1.78	0.84-3.75	1.26	0.7-2.29	4.08	3.22-5.16
**Household members**																
1-2 (ref.)																
3-4	0.46	0.17-1.25	1.34	0.7-2.57	0.74	0.43-1.29	3.53	1.24-10.02	4.68	0.61-35.91	0.43	0.16-1.16	1.05	0.5-2.24	2.08	1.16-3.72
≥5	1.3	0.56-3.03	2.17	1.15-4.08	1.45	0.79-2.64	2.6	0.56-12.07	16654.97	50.7-5471372.19	0.46	0.17-1.22	0.78	0.28-2.21	3.27	2.07-5.18
**Work environment**																
Outdoor (ref.)																
Indoor/Mix	3.43	0.96-12.3	3.38	1.04-10.93	1.56	0.38-6.35	4.44	0.54-36.12	41.23	6.45-263.42	3.57	1.24-10.24	2.53	0.74-8.67	2.66	0.81-8.73
**Smoking status**																
Non-smoker (ref.)																
Current smoker	0.55	0.36-0.84	1.15	0.4-3.35	1.85	0.28-12.3	0.67	0.18-2.55	0.01	0-0.13	0.55	0.15-1.98	0.73	0.19-2.78	0.4	0.12-1.29
**Risk Factors**																
None	1.96	1.24-3.11	1.07	0.77-1.49	3.71	1.37-10.03	0.6	0.06-6.3	6.9	0.54-88.7	0.93	0.6-1.45	0.9	0.44-1.81	0.7	0.4-1.23
One or more (ref.)																
**Vaccine doses**																
Unvaccinated (ref.)																
One dose	2.09	1.47-2.97	1.83	0.92-3.65	1.08	0.49-2.4	0.82	0.16-4.21	15.21	1.04-222.06	2.45	0.98-6.08	5.57	1.87-16.62	2.28	1.26-4.13
Two or more doses	3.37	1.01-11.23	3.94	2.35-6.62	3.35	1.5-7.47	5.75	1.79-18.45	1754.16	47.37-64958.9	2.41	1.27-4.59	3.46	1.41-8.48	2.3	1.29-4.1

*Due to the small sample size, results were not calculated.

Supplementary Table S9. **Regional OR for PT80 for Delta strain multilevel logistic regression, the Dominican Republic, June-October 2021.**

	Yuma		Higuamo		Nordeste/Sur		Noroeste/ El Valle		Norte		Valdesia		Enriquillo		Metropolitana	
	OR	95% CI	OR	95% CI	OR	95% CI	OR	95% CI	OR	95% CI	OR	95% CI	OR	95% CI	OR	95% CI
**Gender**																
Female (ref.)																
Male	0.94	0.47-1.89	0.80	0.54-1.17	2.67	1.17-6.09	0.81	0.2-3.25	0.44	0.13-1.49	0.80	0.6-1.06	0.92	0.57-1.48	1.03	0.64-1.66
Other	0.58	0.12-2.73	0.90	0.21-3.89	*	*	*	*	1.52	0.17-13.77	1.31	0.16-10.42	*		6.45	1.58-26.32
**Age, years**																
05-17	2.80	1.27-6.14	1.19	0.51-2.77	0.39	0.06-2.67	1.33	0.22-8.18	1.20	0.21-6.81	2.12	0.54-8.23	1.89	1-3.6	0.79	0.41-1.51
18-54 (ref.)																
≥55	1.09	0.62-1.92	1.66	1.23-2.23	0.76	0.45-1.28	2.29	0.45-11.72	0.26	0.1-0.67	1.23	0.65-2.35	2.62	0.97-7.05	1.61	1.13-2.28
**Risk Factors Level**																
None/Primary/ Secondary (ref.)																
Tertiary/Technical	0.74	0.16-3.36	0.88	0.56-1.37	0.37	0.18-0.75	7.87	0.42-148.08	0.15	0.02-0.97	1.02	0.46-2.24	0.89	0.52-1.53	1.09	0.71-1.68
**Socioeconomic score**																
0	2.22	0.25-19.43	1.11	0.63-1.96	0.52	0.02-13.58	0.61	0.09-4.19	0.11	0.04-0.28	6.87	1.11-42.37	2.50	1.21-5.13	0.53	0.28-1
1-5 (ref.)																
**Setting**																
Urban (ref.)																
Rural	0.60	0.41-0.88	1.35	0.89-2.07	0.96	0.66-1.41	0.60	0.08-4.7	45.34	12-171.26	0.86	0.64-1.14	1.94	1.51-2.5	1.02	0.88-1.19
**Household members**																
1-2 (ref.)																
3-4	0.48	0.15-1.51	1.08	0.68-1.7	0.51	0.28-0.96	1.62	0.31-8.5	4.37	0.58-33.15	0.47	0.3-0.76	1.32	0.64-2.75	1.52	1.02-2.27
≥5	0.61	0.22-1.68	0.96	0.69-1.34	0.62	0.37-1.04	0.65	0.01-30.75	1.97	0.84-4.64	0.71	0.43-1.16	0.94	0.51-1.73	1.51	1.04-2.18
**Work environment**																
Outdoor (ref.)																
Indoor/Mix	1.18	0.49-2.84	1.44	0.62-3.32	2.28	0.4-12.88	0.70	0.11-4.41	9.66	2.12-44	1.09	0.33-3.59	1.10	0.38-3.21	4.21	1.63-10.87
**Smoking status**																
Non-smoker (ref.)																
Current smoker	0.40	0.1-1.54	0.42	0.1-1.65	3.36	0.98-11.51	0.98	0.11-8.57	0.21	0.02-1.88	0.50	0.12-2.16	0.67	0.33-1.33	0.45	0.2-0.98
**Risk Factors**																
None	1.26	0.86-1.84	1.70	0.9-3.19	0.91	0.59-1.39	0.35	0.09-1.4	0.95	0.27-3.39	0.73	0.43-1.24	0.80	0.5-1.29	0.44	0.31-0.62
One or more (ref.)																
**Vaccine doses**																
Unvaccinated (ref.)																
One dose	4.93	1.75-13.88	2.72	1.21-6.11	2.01	1.21-3.35	0.60	0.02-19.3	62.87	8.66-456.3	1.87	0.9-3.91	2.57	1.42-4.66	2.54	1.43-4.5
Two or more doses	4.79	3.46-6.62	4.15	1.91-9.04	2.31	1.14-4.68	4.66	1.05-20.74	45.28	6.92-296.48	1.97	1.06-3.69	2.31	1.1-4.85	1.94	1.06-3.56

*Due to the small sample size, results were not calculated.

References:

1. Nilles EJ, Paulino CT, de St. Aubin M, Restrepo AC, Mayfield H, Dumas D, et al. SARS-CoV-2 seroprevalence, cumulative infections, and immunity to symptomatic infection – A multistage national household survey and modelling study, Dominican Republic, June–October 2021. The Lancet Regional Health - Americas. 2022;16:100390.
2. Oficina Nacional de Estadistica – Direcion de Estadisticas Demograficas SyA. Anuario de Estadisticas Sociodemograficas, 2021.
